# Innate and Adaptive Immunity to Mucorales

**DOI:** 10.3390/jof3030048

**Published:** 2017-09-05

**Authors:** Harlene Ghuman, Kerstin Voelz

**Affiliations:** School of Biosciences, University of Birmingham, Edgbaston, Birmingham B15 2TT, UK; hxg051@bham.ac.uk

**Keywords:** mucormycosis, spores, hyphae, innate immunity, macrophages, neutrophils, dendritic cells, platelets, adaptive immunity

## Abstract

Mucormycosis is an invasive fungal infection characterised by rapid filamentous growth, which leads to angioinvasion, thrombosis, and tissue necrosis. The high mortality rates (50–100%) associated with mucormycosis are reflective of not only the aggressive nature of the infection and the poor therapeutics currently employed, but also the failure of the human immune system to successfully clear the infection. Immune effector interaction with Mucorales is influenced by the developmental stage of the mucormycete spore. In a healthy immune environment, resting spores are resistant to phagocytic killing. Contrarily, swollen spores and hyphae are susceptible to damage and degradation by macrophages and neutrophils. Under the effects of immune suppression, the recruitment and efficacy of macrophage and neutrophil activity against mucormycetes is considerably reduced. Following penetration of the endothelial lining, Mucorales encounter platelets. Platelets adhere to both mucormycete spores and hyphae, and exhibit germination suppression and hyphal damage capacity in vitro. Dendritic cells are activated in response to Mucorales hyphae only, and induce adaptive immunity. It is crucial to further knowledge regarding our immune system’s failure to eradicate resting spores under intact immunity and inhibit fungal growth under immunocompromised conditions, in order to understand mucormycosis pathogenicity and enhance therapeutic strategies for mucormycosis.

## 1. Introduction

There has been a stark increase in invasive fungal infections over the past two decades [1]. One such infection is mucormycosis, which is caused by species of the Mucorales order. *Rhizopus*, *Mucor* and *Lichtheimia* spp. are responsible for the highest number of reported mucormycosis infections [2]. *Rhizopus oryzae* is the most common causative agent of mucormycosis, followed by *Mucor circinelloides* and *Lichtheimia corymbifera,* which collectively account for around 70% of infections [2,3,4,5,6]. This life-threatening invasive fungal infection carries an extortionate mortality rate, which is estimated at >95% in those with disseminated disease [7]. With medical advances in immunosuppressive treatments and transplantation surgeries, there has inevitably been an increase in the population at risk of mucormycosis [5]. Mucorales commonly establish disease in those with immunocompromised status, with notable pre-disposing risk factors including diabetes mellitus with ketoacidosis (DKA), organ transplantation, chemotherapy, haematological diseases, and elevated serum iron levels [5,8]. The unacceptable high mortality rates associated with mucormycosis are reflective not only of the aggressive nature of the infection and the poor therapeutics currently employed, but also the failure of the human immune system to successfully clear infection. Disease establishment occurs rapidly once filamentous growth has been initiated; it leads to angioinvasion (a hallmark of infection enabling the haematogenous dissemination of disease), thrombosis, and tissue necrosis [9]. Currently, the efficacy of anti-fungal therapeutics, namely liposomal amphotericin B and isavuconazole, is poor (approx. 60–70%), which is pressing great urge on enhancing our current understanding of the Mucorales interaction with the host, in prospect of identifying novel drug targets or treatment strategies [10].

## 2. The Innate Immune Response to Mucorales

Innate immunity is ‘non-specific’ and comprised of physical barriers such as the skin and respiratory mucosa preventing microbial entrance to the host, complement proteins aiding recognition by immune effector cells, and immune effector cells themselves acting in a complex network to combat pathogenic invasion. Mucorales hold the ability to cross physical barriers, most namely the skin, by means of trauma wounds, and the gut by means of ingestion [11,12,13,14]. In the case of pathogen infiltration, the innate immune response acts to cease pathogenic spread, resists the establishment of infection, and triggers the adaptive immune response. Following the successful crossing of physical barriers, Mucorales encounter cells of the innate immune system, including macrophages, neutrophils, and dendritic cells (DC). The ability of Mucorales to establish disease within the human host begins at the failure of the innate immune system to kill fungal spores and halt fungal germination, thus ceasing to limit the expansion of disease [5,15].

Immunosuppressive therapy impairs immune phagocytic effector functions and significantly increases susceptibility to invasive mould infections. For example, macrophage depletion in the zebrafish larvae *Danio rerio* by metronidazole treatment significantly accelerated disease progression and increased mortality in response to Mucorales infection [16]. Likewise, alloxan treatment of rabbits to induce diabetes with acidosis showed delayed leukocyte recruitment to dermal sites of *Rhizopus oryzae* inoculation, an earlier germination of fungal spores, and wider spread of infection [17]. To optimise our understanding of Mucorales pathogenesis, it is necessary to understand the innate immune response to mucormycetes both under immunocompetency and immunocompromisation.

### 2.1. Epithelial Interaction with Mucorales

The first line of defence against Mucorales are the epithelial cells that are encountered at the initial sites of infection, such as alveoli and skin epithelia [18,19]. Patients at risk of invasive mucormycosis exhibit epithelial damage that extends to the basement membrane and exposes extracellular matrix proteins [18]. *R. oryzae* resting spores have been shown to adhere to the basement membrane proteins laminin and type IV collagen [18]. Following adhesion to the basement membrane proteins, Mucorales spores germinate and invade host cells. *R. oryzae*-specific genes encoding lytic enzymes are expressed in those with invasive mucormycosis, which is perhaps indicative of the means of tissue invasion by Mucorales [20].

### 2.2. Macrophage Response to Mucorales

The ability of macrophages to successfully interact with, engulf, degrade, and present pathogenic antigens is key to not only localising infection in its early stages, but also priming the adaptive immune response, which is far more aggressive and specific in the case of pathogenic re-encounter. In order to identify mishaps in this process that may render immunocompromised individuals susceptible to mucormycosis, it is essential to first understand the process under healthy immunity.

During germination, Mucorales sporangiospores exit a dormant, resting phase and undergo both swelling in size and an increase in metabolic activity before commencing filamentous growth [21]. A recent study investigating the phagocytosis of resting spores, swollen spores, and opsonised spores from virulent and attenuated *Lichtheimia corymbifera* strains by murine macrophages, found spores from the virulent strain are more readily phagocytosed than the attenuated strain across all three conditions [22]. Furthermore, resting and swollen spores of the virulent *Lichtheimia* strain were found to be less readily phagocytosed than opsonised spores. Spores of the attenuated *Lichtheimia* strain did not exhibit the same effect of spore opsonisation upon phagocytosis, with opsonised spores being phagocytosed to a similar degree as swollen spores, but more frequently than resting spores [22]. The discovery of virulent *Lichtheimia* spores being phagocytosed to a greater degree than attenuated *Lichtheimia* spores is both interesting and unexpected considering that phagocytosis of pathogens classically leads to the eradication of pathogenic microorganisms. Whilst it has been postulated that virulent Mucorales spores may display this phenomenon to utilise the macrophage as a vector for dissemination, this has yet to be shown.

**Figure 1:**
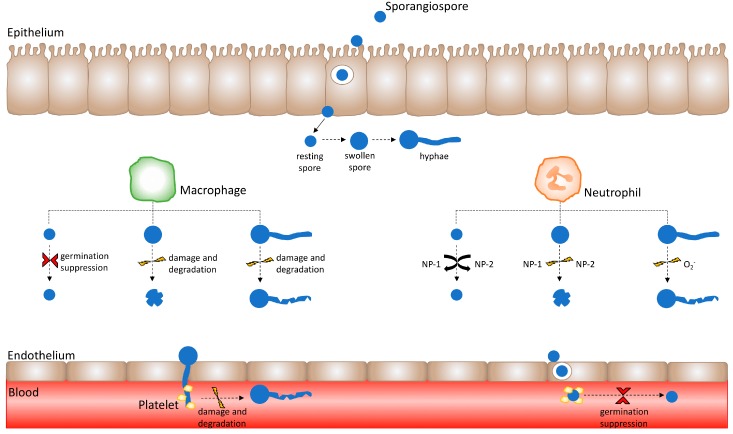
Healthy innate immune effector responses are influenced by the Mucorales spore developmental stage. Immune effector activity varies depending upon the developmental stage of the Mucorales sporangiospore. Following the successful crossing of the epithelium, spores encounter innate immune effectors such as macrophages and neutrophils. Macrophages suppress resting spore germination; however, they are unable to kill resting spores. Conversely, swollen Mucorales spores and hyphae are susceptible to damage and degradation by macrophages. Neutrophil cationic peptides do not exert activity against resting spores, but induce damage upon swollen spores. Moreover, neutrophil superoxide anion (O_2_^−^) release causes damage to hyphae. Having crossed the endothelium layer by means of hyphal invasion and spore internalisation, Mucorales invade the vasculature and enter the bloodstream. Herein, Mucorales come into contact with platelets, whereby platelets adhere to Mucorales spores and hyphae to suppress spore germination as well as cause hyphal damage.

Little consideration has been given to the lack of spore killing by healthy macrophages and its relevance in infection to date. In vitro, rabbit bronchoalveolar macrophage fungicidal activity against *Rhizopus oryzae* spores depends on the developmental stage of spores [23]. Levitz et al. (1986), found the greater the swelling and metabolic activity of spores, the greater the efficacy of macrophage activity against them [23]. In contrast, resting sporangiospores were found to be highly resistant to macrophage activity [23]. Investigating the influence of macrophage activity against Mucorales sporangiospores in healthy murine models has also shown that bronchoalveolar macrophages fail to successfully kill *R. oryzae* spores, although spore germination is suppressed [24] (Figure 1). The *Drosophila melanogaster* model has revealed that the phagocytosis of *R. oryzae* spores is delayed compared with that of *Aspergillus fumigatus* conidia under normal immune status [4]. In addition, the hyphal damage of *R. oryzae* is significantly reduced compared with that of *A. fumigatus* [4]. Collectively, these elucidations indicate that the healthy human host is not wholly effective in killing resting Mucorales spores. Although reported cases of mucormycosis in immunocompetent hosts are rare, preliminary revelations that healthy macrophages are incapable of killing Mucorales spores offers worthy reason for understanding why this is so [25,26,27]. The classical understanding of pathogenic clearance appears not to be fully applicable to our understanding of Mucorales clearance, and highlights the need for enhancing our knowledge of Mucorales–macrophage interactions under normal immune conditions.

The literature on Mucorales–macrophage interactions under normal vs. immunocompromised states is limited. However, studies have elucidated differences in macrophage responses to Mucorales between the two. Survival studies looking at mucormycosis in healthy vs. immunocompromised model organisms, such as zebrafish and *Drosophila*, show that immunosuppressive treatment significantly increases mortality [4,16]. In vivo studies on the effect of immunosuppression upon macrophage–Mucorales interactions confirm that phagocytic activity is considerably impaired when compared with their activity under a healthy immune state [4,28]. Cortisone treatment, rendering murine models immunocompromised, revealed not only the inability of macrophages to kill *R. oryzae* spores, but also the failure to inhibit spore germination [28]. Dexamethasone treatment of *Drosophila* mimics that of human corticosteroid treatment and results in immunosuppression. Under such treatment, *D. melanogaster* displays impaired phagocytic efficacy against *R. oryzae* compared with that observed in healthy flies [4]. Moreover, hyphal damage is further reduced and phagocytosis of *Rhizopus* spores is void altogether [4].

Understanding mucormycete–macrophage interactions under immunocompetency versus immunocompromisation is crucial for understanding how Mucorales take advantage of those with dampened immunity. The inability of macrophages to inhibit Mucorales spore germination under immunocompromised conditions appears to be, in part, fundamental to the establishment of mucormycosis. This stark difference in macrophage response to Mucorales, both with regards to spore developmental stage and morphological form, may elucidate how these opportunistic fungal pathogens evade macrophage activity and establish disease so aggressively.

### 2.3. Neutrophil Response to Mucorales

Neutrophils are the most abundant type of leukocytes found in the blood, and are rapidly recruited to the site of pathogenic infection [29,30,31]. The ability of these innate immune cells to phagocytose and destroy pathogens in a non-specific and rapid manner is crucial in combatting pathogenic invasion [31]. Neutrophils not only destroy pathogens via an array of means, such as neutrophil cationic peptides and oxidative bursts, but also play a key role in mediating acute inflammation and maintaining haemostasis by infiltrating infected sites and undergoing timely apoptosis [30,31,32].

The neutrophil–Mucorales interaction is of great interest when considering mucormycosis, especially due to its associated risk factor, neutropenia [33,34]. Under healthy immune conditions, neutrophils are shown not to be readily recruited to resting Mucorales spores in the lungs of intranasally infected mice [35]. Intranasal inoculation of swollen *Rhizopus* spores; however, sees a marked increase in both neutrophil recruitment and inflammation [34]. Moreover, in vitro chemotactic studies show that swollen *Rhizopus* spores produce neutrophil chemotactic factors, but resting spores do not [35]. Neutrophils exhibit fungicidal activity mediated by the production of cationic peptide activity [36]. As was observed with macrophage activity against Mucorales spores, it was found that the *R. oryzae* spore developmental stage influences the efficacy of neutrophil-killing activity. Neutrophil cationic peptides NP-1 and NP-2 have redundant activity against resting *R. oryzae* spores; however, against swollen *R. oryzae* spores, killing by NP-1 and NP-2 is effective (Figure 1) [24]. *Rhizopus* hyphae have been shown to induce the expression of *tlr2* mRNA in human polymorphonuclear neutrophils, which indicates that TLR2 contributes as a pattern recognition receptor towards Mucorales recognition by these leukocytes [37]. Following exposure to *Rhizopus* hyphae, neutrophil expression of pro-inflammatory genes such as *tnf-α* and *il-1b* is noted, which implies neutrophil activation in response to Mucorales [37]. Neutrophils are capable of causing *R. oryzae* hyphal damage, albeit to an attenuated degree compared with the damage they cause to *A. fumigatus* hyphae [37,38]. Reduced hyphal damage to mucormycetes by neutrophils is associated with a dampened oxidative burst, as seen by reduced superoxide anion release [38].

Hyperglycemia and ketoacidosis both impair phagocytic effector functions [39]. Comparisons drawn between *R. oryzae* hyphae treated with normal human serum versus DKA serum indicate the strong influence of hyperglycaemia and low pH on the killing of Mucorales hyphae by neutrophils [38]. Not only is neutrophil migration to hyphae considerably impaired under hyperglycaemic and ketoacidosis conditions, effective hyphal killing by these phagocytes is also reduced [38].

The influence of Mucorales spore developmental stage on the efficacy of neutrophil combat under immunocompetency coincides with that concerning macrophages. Since resting spores are able to evade neutrophil encounters by bearing negligible chemotactic properties, they have an advantage over the healthy host. Whilst neutrophils are capable of killing swollen Mucorales spores and hyphae, the degree of damage and destruction is significantly lower when compared with that of *Aspergillus*. This shortfall in neutrophil phagocytic effect is exaggerated further under immunocompromised conditions such as hyperglycaemia. As neutrophils are one of the early innate immune effectors that Mucorales are likely to encounter, and as these leukocytes play a key role in acute inflammation, enhancing our current understanding of Mucorales pathogenesis enabling neutrophil evasion could prove paramount in combatting disease.

### 2.4. Endothelial Interaction with Mucorales

Angioinvasion is a hallmark of mucormycosis. Penetration of the endothelial lining of vasculature results in thrombosis and tissue necrosis, and is the process that enables Mucorales to disseminate haemotogenously. *R. oryzae* adheres directly to endothelial cells, and induces injury through internalisation. Entry to endothelial cells is mediated by the endothelial cell surface receptor GRP78, the expression of which is greatly enhanced when under DKA and hyperglycaemic conditions [5,19]. Iron chelation therapy in mouse models showed that *Rhizopus* was unable to successfully invade endothelial cells, and highlighted that Mucorales internalisation is host iron dependent [19].

### 2.5. Platelet-Mucorales Interaction

Platelets have been classically affiliated with haemostasis, thrombosis, and inflammation [40]. Recently, platelets have been identified as key innate immune effectors. These small, anuclear thrombocytes bear antimicrobial properties that are mediated via granular release of platelet antimicrobial peptides such as platelet factor 4 (PF-4), as well as display chemotactic properties toward phagocyte recruitment, mediated via the release of cytokines such as IL-1b [40,41].

As thrombosis is a hallmark of mucormycosis, the role of platelets in the innate immune response to Mucorales is of great interest. In vitro studies show that platelets adhere to both Mucorales spores and hyphae as well as induce platelet activation and granule release [42]. Platelet interaction significantly inhibits fungal germination, reduces *Rhizopus*, *Mucor*, *Lichtheimia* and *Rhizomucor* hyphal growth, and induces hyphal damage (Figure 1) [42].

The ability of platelets to inhibit Mucorales germination suggests that these innate immune effectors play a beneficial role. Contrarily, excessive thrombosis results in thrombocytopenia, and makes surgical intervention for diagnosis and treatment undesirable [43]. Whilst preliminary research has identified that platelets play a direct role in immunity against Mucorales, the underlying mechanisms remain to be identified. Furthermore, the effects of immunosuppression and immunocompromisation on platelet–Mucorales interactions have not been divulged.

### 2.6. Dendritic Cell–Mucorales Interaction 

Dendritic cells (DCs) play a pivotal role in the host response to pathogenic invasion by means of acting as the major antigen-presenting cell for adaptive immune effectors and triggering the adaptive immune system [44]. In a healthy immune setting and upon pathogen recognition, DCs phagocytose pathogens, secrete pro-inflammatory cytokines, and present pathogenic antigens to T and B lymphocytes [45,46,47].

**Figure 2:**
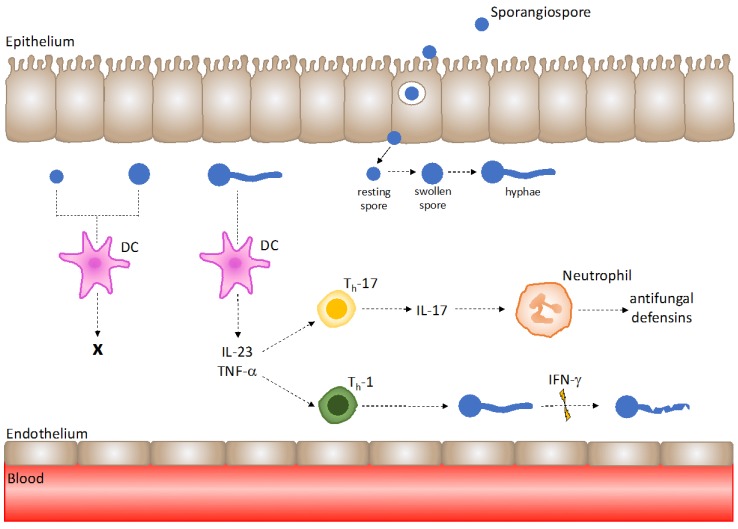
*Mucorales hyphae are recognised by* *dendritic cells (DCs) to trigger T-helper cell responses under healthy host immunity.* DCs are not activated by Mucorales spores; however, exposure to hyphae results in DC activation and the robust release of IL-23 and TNF-α. IL-23 promotes T_h_-17 responses, and TNF-α upregulates T_h_-1 responses. IL-17 production by T_h_-17 cells aids neutrophil recruitment and antifungal defensin release, and IFN-γ secretion by T_h_-1 cells exhibits Mucorales hyphal damage.

The DC–Mucorales interaction is less well researched than those of other immune effectors and mucormycetes. An in vitro study showed that DC activation does not occur in response to *Rhizopus* spores; however, hyphae were shown to induce a strong DC release of IL-23, which is known to drive T_h_-17 responses, and TNF-α, which is known to upregulate T_h_-1 responses (Figure 2) [48,49]. β-glucanase treatment of *Rhizopus* hyphae showed that the fungal cell wall component β-glucan is essential for IL-23 production and T_h_-17 responses by DCs [48]. Furthermore, the inhibition of the DC β-glucan receptor dectin-1 abolished IL-23 production by DCs, elucidating that DC activation occurs in response to Mucorales surface β-glucan, and is dectin-1-dependent [48].

Similar to macrophages and neutrophils, the response of DCs differs significantly depending upon the stage of germination of the Mucorales sporangiospore. Literature is limited regarding DC–Mucorales interaction; however, it is apparent that healthy DCs fail to recognise resting Mucorales spores. DCs are key drivers of the adaptive immune response, and thus investigations of DC–Mucorales interactions should be extended to encompass the effect of immunosuppression on their efficacy.

### 2.7. Natural Killer Cell Response to Mucorales 

Natural killer (NK) cells play a regulatory and cytotoxic role in the innate immune response to pathogens [50]. Mucorales hyphae penetrate epithelial and endothelial tissue, causing extensive tissue damage. As natural killer cells are responsible for limiting tissue damage by means of inducing cell cytoxicity, the role of these lymphocytes in mucormycosis should not be disregarded. NK cell activity is highly dependent upon signalling from macrophages, DCs, and T cells [50]. Cytokines such as IL-12 and type-1 interferon (IFN) are potent NK cell activators and mediate the interplay between macrophages and NK cells, which frequently co-localise at sites of infection and tissue damage. Whilst traditionally associated with handling tumour cells, NK cells have also been suggested to play a role in minimising exacerbated macrophage activity during infection [51].

The role of NK cells in mucormycosis is an understudied area of research, yet it has been shown that *Rhizopus* spores do not activate NK cells and are resistant to their fungicidal activity [51]. In contrast, *Rhizopus* hyphae activate NK cells and are damaged by human natural killer cells through the release of perforin [51]. However, *Rhizopus* hyphae also exhibit the capacity to suppress secretion of immunoregulatory molecules by NK cells, such as interferon-γ (IFN-γ) and RANTES, which suggests that Mucorales hyphae have an immunosuppressive effect upon NK cells [7,51].

## 3. The Adaptive Immune Response to Mucorales

The adaptive immune system plays a secondary role in handling invasive fungal infections caused by Mucorales. Initially, T cells were believed to have little significance in the immune response due to T-cell deficiency bearing no influence on an individual’s risk to Mucorales infection [52]. The implication of DCs inducing T_h_-17 responses, however, suggests that T-cell responses to mucormycetes are activated and worthy of investigation.

### T-Cell Responses to Mucorales

T_h_ cells are noted to play a key role in the clearance of pathogenic fungi, which is mediated by the secretion of distinct cytokines [53]. Interferon-γ producing T_h_-1 cells confer protective immunity against fungi, whereas T_h_-2 responses increase susceptibility to fungal infections [53]. T_h_-17 cells are named after their robust IL-17 production, and have also been identified and implicated in mucosal immunity against fungi [53].

In vitro stimulation of T cells by *Rhizopus oryzae* showed the generation of T_h_-17 cells [48]. T_h_-17 cell production of IL-17 has a profound impact on neutrophil activity by acting as a chemoattractant and inducing the production of antifungal defensins by neutrophils (Figure 2) [53]. T_cyt_-cells were not found to be generated against *R. oryzae* [48].

A study investigating mucormycete-specific T-cells in patients with invasive mucormycosis found that Mucorales-specific T-cells belonging to both CD4^+^ and CD8^+^ subsets were present during infection. Furthermore, the investigation of Mucorales-specific T-cell cytokine profiles showed IL-4 (T_h_-2 cytokine), IFN-γ and IL-10 (T_h_-1 cytokine) to be most abundantly produced, followed by IL-17 [54]. Additionally, IFN-γ producing T-cells were shown to induce Mucorales hyphal damage (Figure 2) [54].

Research upon T-cell involvement in immunity against Mucorales is sparse, though preliminary findings highlight the induction of a T_h_-17 response which may strengthen the neutrophilic attack on mucormycetes. Conducting in vivo investigations of T-cell responses to Mucorales under healthy and compromised host immunity would be desirable to enhance our understanding of how T-cells interact with this fungal pathogen.

## 4. Conclusions

Invasive fungal infections are increasingly becoming a concern with the rise in fatal cases seen as of late. Mucormycosis cases have been increasing globally, yet the efficacy of current therapeutics remains unacceptably poor at around 60–70%. Understanding the pathogenicity of invasive fungal infection causing Mucorales is paramount in combatting the high fatality rates associated with fungi of this order. Mucorales are able to evade pathogenic clearance by the host immune system and undergo germination within the host, whereby hyphae formation allows for angioinvasion, dissemination, and irreparable tissue damage. The inability of a compromised host immune system to suppress Mucorales germination is critical in enabling mucormycetes to establish infection, which upon widespread dissemination is virtually impossible to resolve.

Mucorales are incredibly efficient at overcoming intact epithelia and endothelia, although the exact nature of facilitation is unknown. Elucidating the molecular underpinnings of endothelial and epithelial invasion could prove fruitful in identifying novel drug targets that may cease Mucorales in its tracks before it encounters the innate immune system.

The developmental stage of the sporangiospore influences the efficacy of macrophage phagocystosis and killing upon it, with swollen mucormycete spores being less successful at surviving the mechanisms of macrophage killing. Moreover, once in its hyphal form, *R. oryzae* succumbs to macrophage fungicidal damage. Similar to macrophages, neutrophils and NK cells are redundant in fungicidal activity against resting Mucorales spores; however, these phagocytes are able to effectively kill swollen sporangiospores and hyphae alike. Dendritic cells play a crucial role in mediating the initiation of an adaptive immune response to Mucorales, and their response again differs significantly depending upon the developmental stage of Mucorales spores. Resting *Rhizopus* spores fail to induce robust IL-23 release by DCs, whereas exposure to *Rhizopus* hyphae sees a dramatic increase in IL-23 and TNF-α production.

The role of the adaptive immune system in response to Mucorales is understudied with respect to that of the innate immune system. *R. oryzae* induces a strong T_h_-17 response that is noted by the robust secretion of IL-17, which plays a key role in recruiting and initiating neutrophil responses to *R. oryzae*. Furthermore, IFN-γ producing T-cells have been shown to damage Mucorales hyphae. Whilst in vitro study has elucidated the activation of T-cells in response to Mucorales, the influence of spore developmental stage is not well addressed, and in vivo research is lacking in the current literature.

Enhancing our understanding of the immune response to Mucoralean fungi is crucial in identifying how these pathogenic species evade immune defence mechanisms, and improving current therapeutics for mucormycosis. The delicate differences in Mucorales clearance by immune cells, as seen between the different developmental stages of Mucorales spores and hyphae, is noteworthy and indicative of how this fungal pathogen does not succumb to immune clearance.
